# Antimicrobial resistance determinants and susceptibility profiles of pneumococcal isolates recovered in Trinidad and Tobago^☆^

**DOI:** 10.1016/j.jgar.2017.08.004

**Published:** 2017-12

**Authors:** Paulina A. Hawkins, Patrick E. Akpaka, Michele Nurse-Lucas, Rebecca Gladstone, Stephen D. Bentley, Robert F. Breiman, Lesley McGee, William H. Swanston

**Affiliations:** a Emory University, Atlanta, GA, USA; b US Centers for Disease Control and Prevention (CDC), Atlanta, GA, USA; c The University of the West Indies, St. Augustine, Trinidad and Tobago; d Wellcome Trust Sanger Institute, Cambridge, UK

**Keywords:** *Streptococcus pneumoniae*, Antimicrobial resistance, Whole-genome sequencing

## Abstract

**Objectives:**

In Latin America and the Caribbean, pneumococcal infections are estimated to account for 12 000–18 000 deaths, 327 000 pneumonia cases, 4000 meningitis cases and 1229 sepsis cases each year in children under five years old. Pneumococcal antimicrobial resistance has evolved into a worldwide health problem in the last few decades. This study aimed to determine the antimicrobial susceptibility profiles of pneumococcal isolates collected in Trinidad and Tobago and their associated genetic determinants.

**Methods:**

Whole-genome sequences were obtained from 98 pneumococcal isolates recovered at several regional hospitals, including 83 invasive and 15 non-invasive strains, recovered before (*n* = 25) and after (*n* = 73) introduction of pneumococcal conjugate vaccines (PCVs). A bioinformatics pipeline was used to identify core genomic and accessory elements conferring antimicrobial resistance phenotypes, including β-lactam non-susceptibility.

**Results and discussion:**

Forty-one isolates (41.8%) were predicted as resistant to at least one antimicrobial class, including 13 (13.3%) resistant to at least three classes. The most common serotypes associated with antimicrobial resistance were 23F (*n* = 10), 19F (*n* = 8), 6 B (*n* = 6) and 14 (*n* = 5). The most common serotypes associated with penicillin non-susceptibility were 19F (*n* = 7) and 14 (*n* = 5). Thirty-nine isolates (39.8%) were positive for PI-1 or PI-2 type pili: 30 (76.9%) were PI-1+, 4 (10.3%) were PI-2+ and 5 (12.8%) were positive for both PI-1 and PI-2. Of the 13 multidrug-resistant isolates, 10 belonged to globally distributed clones PMEN3 and PMEN14 and were isolated in the post-PCV period, suggesting clonal expansion.

## Highlights

• AWGS-based approach can accurately and reliably predict antimicrobial phenotypes.
• Observed rates of non-susceptibility to SXT and erythromycin were lower than reported for other countries in the region.
• In contrast,the proportion of β-lactam non-susceptibility was higher compared with other countries in the region.
• Multidrug resistance remains low but appears to be expanding clonally following introduction of pneumococcal vaccines.
• This clonal expansion is driven by the 19F-CC156 and 19A/F-CC236 lineages.

## 1 Introduction

Infections caused by *Streptococcus pneumoniae* include diseases such as meningitis, bacteraemia and pneumonia as well as less severe conditions such as sinusitis and otitis media. The World Health Organization (WHO) estimated that pneumococcal infections caused 476 000 (5%) deaths globally among human immunodeficiency virus (HIV)-negative children under 5 years of age during 2008 [1]. In Latin America and the Caribbean, pneumococcal infections were estimated to account for 12 000–18 000 deaths, 327 000 cases of pneumonia, 4000 cases of meningitis and 1229 cases of sepsis each year in children under five years old [2].

The first pneumococcal conjugate vaccine (PCV) covered seven serotypes (PCV7: serotypes 14, 6B, 19F, 23F, 4, 9V and 18C) and was licensed in 2000, followed by PCV10 (PCV7 serotypes plus 1, 5 and 7F) in 2009, and PCV13 (PCV10 serotypes plus 3, 6A and 19A) in 2010. In the Caribbean, as well as in other regions, vaccine uptake has been variable. PCV7 was introduced into the national immunisation programme (NIP) in Trinidad and Tobago in March 2010 for infants at risk of pneumococcal disease, mainly those with immune deficiencies and other chronic diseases. Prior to 2010, PCV7 was only available in the private sector [3]. PCV10 was introduced into the NIP in 2011 for all children aged <2 years [3], [4], [5] and was replaced by PCV13 in August 2015. As of December 2014, vaccine coverage with PCV10 was reported to be 95% [6].

From the beginning of the antibiotic era to the mid-1970s, *S. pneumoniae* remained uniformly susceptible to all classes of antibiotics that were active against it, with the exception of tetracycline. In the ensuing decades, resistance of pneumococci to a variety of antimicrobials has evolved into a worldwide health problem [7]. A 2004 report by the SENTRY surveillance program showed that penicillin non-susceptibility rates were as high as 25% among pneumococcal isolates, with penicillin-non-susceptible isolates presenting higher rates of multidrug resistance [8].

In the Caribbean region, infections due to penicillin-resistant pneumococci have been reported in hospitals and community settings, but limited data are available to estimate patterns of drug resistance [9]. The aim of this study was to determine the antimicrobial susceptibility profiles of 98 pneumococcal isolates collected in Trinidad and Tobago from invasive and non-invasive sites and their associated genetic determinants.

## 2 Materials and methods

A total of 98 pneumococcal isolates recovered at several regional hospitals in Trinidad and Tobago were included in the study, including 83 invasive and 15 non-invasive strains. All pneumococcal isolates (*n* = 73) obtained from routine clinical specimens at the five major public hospitals in Trinidad and Tobago during the period 2011–2013 were included in this study. A number of historical pneumococcal isolates (*n* = 25) from clinical specimens that were collected between 1997–2010 were also included in the analysis; most of the strains were collected from three regional hospitals prior to the start of the SIREVA (Sistema regional de vacunas) project in Trinidad and Tobago. Serotyping and multilocus sequence typing (MLST) results from these 98 isolates have been published previously [10]. Minimum inhibitory concentrations (MICs) were determined using the broth microdilution method as specified by Clinical and Laboratory Standards Institute (CLSI) guidelines [11]. Penicillin susceptibility, intermediate resistance and resistance were defined as MICs of ≤0.06, 0.12–1.0 and ≥2.0 mg/L, respectively. Cefotaxime and/or ceftriaxone susceptibility, intermediate resistance and resistance were defined as MICs of ≤0.5, 1.0 and ≥2.0 mg/L, respectively. For amoxicillin, susceptibility, intermediate resistance and resistance were defined as MICs of ≤0.12, 0.25–1 and ≥2.0 mg/L, respectively. For meropenem, susceptibility, intermediate resistance, resistance and high-level resistance were defined at ≤0.25, 0.5, 1.0 and ≥2.0 mg/L, respectively. For previously unreported penicillin-binding protein (PBP) types, MICs against penicillin and cefotaxime were determined by Etest (bioMérieux, Marcy-l’Étoile, France).

*S. pneumoniae* strains were cultured on BD™ Trypticase™ Soy Agar II with 5% sheep blood (Beckton Dickinson, Heidelberg, Germany) and incubated overnight at 37 °C in 5% CO_2_. Genomic DNA was then extracted manually using a modified QIAamp^®^ DNA Mini Kit (QIAGEN, Inc., Valencia, CA) protocol. Whole-genome sequencing (WGS) was performed at the Sanger Institute using an Illumina HiSeq 2500 system (Illumina Inc., San Diego, CA) as part of the Global Pneumococcal Sequencing Project (http://www.pneumogen.net) and the resulting data were submitted to the European Nucleotide Archive (accession nos. are given in Supplementary Table S1). Sequences were analysed using the *Streptococcus* laboratory pneumococcal typing pipeline of the US Centers for Disease Control and Prevention (CDC) [12], [13] to identify core genomic alterations and accessory elements that confer antimicrobial resistance phenotypes as well as pilus genes (https://github.com/BenJamesMetcalf/Spn_Scripts_Reference). Non-susceptibility to β-lactams was predicted based on three key PBP transpeptidase domain sequences or PBP types [13], [14], which correlate with recorded MICs for each of the six β-lactams (penicillin, amoxicillin, ceftriaxone, cefotaxime, cefuroxime and meropenem) (http://www.cdc.gov/streplab/mic-tables.html). Contingency tables and a *χ*^2^ test (or Fisher’s exact test) were used to determine the significance of associations (at *α* = 0.05).

## 3 Results and discussion

### 3.1 Antimicrobial resistance

The capability of a WGS-based approach to accurately and reliably predict antimicrobial phenotypes has been previously shown to be an adequate substitute for broth dilution susceptibility testing [13], [14]. By a WGS-based assessment of resistance, 34 (34.7%) of the 98 isolates in this study were predicted to be non-susceptible to trimethoprim/sulfamethoxazole (SXT), 18 (18.4%) resistant to erythromycin, 17 (17.3%) non-susceptible to β-lactams, 9 (9.2%) resistant to tetracycline, 2 (2.0%) resistant to chloramphenicol and 1 (1.0%) resistant to rifampicin. These results were in agreement with the MICs determined by broth microdilution (Table 1). Overall, 41 isolates (41.8%) were predicted to be resistant to at least one antimicrobial class, including 13 (13.3%) resistant to at least three classes [i.e. multidrug-resistant (MDR)]. Before PCV7/10 introduction, 28.0% (7/25) of the isolates were resistant to at least one antimicrobial class; this proportion increased to 46.6% (34/73) in the post-PCV period (*p* = 0.06). The proportion of MDR isolates increased from 4.0% (1/25) to 16.4% (12/73) after PCV7/10 introduction (*p* = 0.06).

**Table 1:** Non-susceptibility predicted by whole-genome sequencing versus non-susceptibility determined from observed minimum inhibitory concentrations (MICs).

Resistance determinant (s)	Non-susceptible		Observed MIC range (mg/L)					
	Predicted	Observed	ERY	CLI	TET	SXT	CHL	RIF
*folA* + *folP*^a^	19	19				≥4		
*folP* only^a^	15	15				1–2		
*ermB* only	2	2	>32	>2				
*mef* only	11	10^b^	0.12–16	0.06–0.12				
*ermB* + *mef*	5	5	>32	>2				
*tetM*	9	9			>8			
*cat*	2	2					>8	
*rpoB* (H499Y)	1	1						>2

ERY, erythromycin; CLI, clindamycin; TET: tetracycline; SXT, trimethoprim/sulfamethoxazole; CHL, chloramphenicol; RIF, rifampicin.

a One to two codon insertions within the *folP* gene (at nucleotides 171, 176, 177, 178, 180, 185, 186 or 195) result in an intermediate phenotype (MIC 1–2 mg/L) against SXT; when combined with the *folA* substitution I100L, they result in a resistant phenotype (MIC ≥ 4 mg/L).

b One isolate was susceptible to erythromycin despite being *mef*-positive (MIC = 0.12 mg/L).

A total of 37 PBP allele combinations (PBP types) were identified among these isolates, 11 of them novel; 8 (21.6%) of these combinations (3 of them novel) were associated with non-susceptibility to penicillin (MIC ≥ 0.12 mg/L). Identifying new allele combinations was expected as the database used for analyses mostly contains isolates from the USA. Of the 17 isolates that were predicted as non-susceptible to β-lactams, all were predicted as penicillin non-susceptible (PNS), 9 were predicted as non-susceptible to ceftriaxone, cefuroxime, cefotaxime and meropenem (in addition to penicillin), and 3 were predicted as non-susceptible to all six of the β-lactams tested.

Of the 34 isolates predicted as non-susceptible against co-trimoxazole, all contained one to two codon insertions within the *folP* gene (intermediate phenotype, MIC 1–2 mg/L), whilst 19 (55.9%) also contained the I100L substitution in *folA* (resistant phenotype, MIC ≥ 4 mg/L). Of the 18 isolates predicted as resistant to erythromycin, 11 (61.1%) were positive for *mef* alone, 2 (11.1%) for *ermB* alone and 5 (27.8%) for *ermB* + *mef*; the latter 7 isolates containing *ermB* were also predicted as resistant to clindamycin. In addition, nine isolates were positive for *tetM* and two isolates for the *cat* gene. One isolate contained a change in the *rpoB* gene (H499Y) and was resistant to rifampicin by broth microdilution (MIC > 2 mg/L).

The observed rate of non-susceptibility against co-trimoxazole (34.7%) was lower than what has been reported for other countries in the region [15] such as Venezuela (100%) and the Dominican Republic (65%); the rate of resistance to erythromycin (18.4%) was similarly lower (compared with 45% in Venezuela and 20% in Dominican Republic). In contrast, the proportion of β-lactam non-susceptibility (17.3%) was higher among these isolates than among isolates from the Dominican Republic (9.6%), but was similar to that observed among isolates recovered in Venezuela (18.2%).

The most common serotypes associated with antimicrobial resistance were 23F (*n* = 10), 19F (*n* = 8), 6 B (*n* = 6) and 14 (*n* = 5). The most common serotypes associated with PNS were 19F (*n* = 7) and 14 (*n* = 5). The 13 MDR belonged to only four different clonal complexes/sequence types (CCs/STs) and four serotypes, mostly CC156 (global clone PMEN3) and CC236 (global clone PMEN14), suggesting a clonal expansion following PCV7/10 introduction (Table 2).

**Table 2:** Clonal complexes (CCs) and sequence types (STs) associated with multidrug-resistant pneumococcal isolates.

CC/ST	*n*	Serotype (*n*)	PBP types (*n*)	Resistance phenotype
CC156 (PMEN3)	4	19F (4)	15:12:36 (3) 121:12:36 (1)	SXT, ERY, PEN, CTX, CRO, CFX, MEM
CC236 (PMEN14)	6	19F (3)	13:16:47 (2) 119:16:47 (1)	SXT, ERY, CLI, TET, PEN, CTX, CRO, CFX, MEM
		19A (3)	13:11:16 (3)	SXT, ERY, CLI, TET, PEN, AMX, CTX, CRO, CFX, MEM
ST554	2	14	120:16:80 (14)	ERY, CLI, TET, CHL, PEN
ST490	1	6A	Susceptible	SXT, ERY, RIF

PBP, penicillin-binding protein; SXT, trimethoprim/sulfamethoxazole; ERY, erythromycin; PEN, penicillin; CTX, cefotaxime; CRO, ceftriaxone; CFX, cefuroxime; MEM, meropenem; CLI, clindamycin; TET, tetracycline; AMX, amoxicillin; CHL, chloramphenicol; RIF, rifampicin.

### 3.2 Pilus genes

In *S. pneumoniae*, pili are encoded by two different pathogenicity islets, type 1 (PI-1) and type 2 (PI-2). The Pilus Islet-1, particularly the RrgA subunit, has been shown to not only contribute to adherence and virulence but to also stimulate the host inflammatory response [16]. The Pilus Islet-2 has also been shown to contribute to adherence but in a less effective manner than PI-1 [17]. Overall, 39 isolates (39.8%) were positive for PI-1 or PI-2 type pili (inferred by detection of *rrgA* or *pitB* pilus subunit genes): 30 (76.9%) of them were solely PI-1+, 4 (10.3%) were PI-2+ and 5 (12.8%) were positive for both PI-1 and PI-2.

Consistent with previous reports [18], the presence of PI-1 or both of the pilus loci was associated with certain CCs and serotypes (Table 3) and, in consequence, with antimicrobial susceptibility profiles. Of the 30 PI-1-only isolates, 11 (36.7%) belonged to CC156 (PMEN3) and serotypes 9V, 14 and 19F; all serotype 9V isolates were susceptible to all antibiotics tested, all serotype 14 were PNS, and all 19F isolates were MDR. In addition, 7 (23.3%) PI-1-only isolates belonged to ST138 (5 of them serotype 6B) and 5 (16.7%) to CC145 (all serotype 6B); the ST138 isolates were susceptible to all drugs, whilst 3 of the CC145 isolates were non-susceptible to co-trimoxazole (intermediate phenotype). All five PI-1 + PI-2 isolates belonged to CC236 (PMEN14) and serotypes 19A/19F and were MDR. Three (75.0%) of the four PI-2-only isolates belonged to CC62 (serotype 11A) and the remaining isolate to ST191 (serotype 7F); all four were susceptible to all antimicrobials tested.

**Table 3:** Sequence types/clonal complexes (ST/CC) and serotypes associated with the presence of pilus loci.

ST/CC	*n*	PI-1	PI-2	PI-1 + PI-2	Associated serotypes (*n*)
CC156	11	11			19F (4), 9V (4), 14 (3)
ST138	7	7			6B (5), 6A (1), 19F (1)
CC145	5	5			6B (5)
CC236	6	1		5	19A (3), 19F (3)
ST695	2	2			19A (2)
CC490	2	2			6A (1), 19F (1)
ST205	1	1			4 (1)
ST497	1	1			6B (1)
CC62	3		3		11A (3)
ST191	1		1		7F (1)

PI-1, pilus locus 1; PI-2, pilus locus 2.

In conclusion, this study offers a snapshot of the antimicrobial resistance profiles and genetic resistance determinants among 98 pneumococcal isolates recovered in Trinidad and Tobago, adding to the limited body of data available for the Caribbean region. The observed rates of resistance were similar to those reported for neighbouring Caribbean countries. Multidrug resistance remains low but appears to be expanding clonally following PCV7/10 introduction, driven by the 19F-CC156 and 19A/F-CC236 lineages. Thus, the introduction of PCV13 will likely have a marked impact on pneumococcal multidrug resistance in Trinidad and Tobago.

## Competing interest

None declared.

## Funding

Isolates were characterised as part of the Global Pneumococcal Strain Bank established with funding from PATH and currently housed at the US Centers for Disease Control and Prevention (CDC) (https://www.cdc.gov/streplab/global-pneumo-strain-bank.html). Whole-genome sequencing was performed as part of the Global Pneumococcal Sequencing Project, funded by the Bill and Melinda Gates Foundation [grant no. OPP1034556]. The funding sources had no involvement in the study design, the collection, analysis or interpretation of data, the writing of the report, or the decision to submit the article for publication.

## Ethical approval

Not required.
