# Supplementary material for: Antimicrobial resistance determinants and susceptibility profiles of pneumococcal isolates recovered in Trinidad and Tobago
Source: J Glob Antimicrob Resist. 2017 Dec;11:148–51. doi: 10.1016/j.jgar.2017.08.004 (PMC5711790; doi:10.1016/j.jgar.2017.08.004)
Supplement: Supplementary file 1 [file mmc1.docx]

**Supplemental Table S1**. European Nucleotide Archive accession numbers.

| **Isolate name** | **Accession No** |
| --- | --- |
| GPS_P6441 | ERR751155 |
| GPS_P6442 | ERR751156 |
| GPS_P6443 | ERR751157 |
| GPS_P6444 | ERR751158 |
| GPS_P6445 | ERR751159 |
| GPS_P6446 | ERR751160 |
| GPS_P6447 | ERR751161 |
| GPS_P6448 | ERR751162 |
| GPS_P6449 | ERR751163 |
| GPS_P6450 | ERR751164 |
| GPS_P6451 | ERR751165 |
| GPS_P6452 | ERR751166 |
| GPS_P6453 | ERR751167 |
| GPS_P6454 | ERR845789 |
| GPS_P6455 | ERR751168 |
| GPS_P6456 | ERR751169 |
| GPS_P6457 | ERR751170 |
| GPS_P6458 | ERR751171 |
| GPS_P6459 | ERR751172 |
| GPS_P6460 | ERR751173 |
| GPS_P6461 | ERR751174 |
| GPS_P6462 | ERR751175 |
| GPS_P6463 | ERR751176 |
| GPS_P6464 | ERR751177 |
| GPS_P6465 | ERR751178 |
| GPS_P6466 | ERR751179 |
| GPS_P6467 | ERR751180 |
| GPS_P6468 | ERR751181 |
| GPS_P6469 | ERR751182 |
| GPS_P6470 | ERR751183 |
| GPS_P6471 | ERR751184 |
| GPS_P6472 | ERR751185 |
| GPS_P6473 | ERR751186 |
| GPS_P6474 | ERR751187 |
| GPS_P6475 | ERR751188 |
| GPS_P6476 | ERR751189 |
| GPS_P6477 | ERR845790 |
| GPS_P6478 | ERR751190 |
| GPS_P6479 | ERR751191 |
| GPS_P6480 | ERR751192 |
| GPS_P6481 | ERR751193 |
| GPS_P6482 | ERR751194 |
| GPS_P6483 | ERR751195 |
| GPS_P6484 | ERR751096 |
| GPS_P6485 | ERR751097 |
| GPS_P6486 | ERR751098 |
| GPS_P6487 | ERR751099 |
| GPS_P6488 | ERR751100 |
| GPS_P6489 | ERR751101 |
| GPS_P6490 | ERR751102 |
| GPS_P6491 | ERR876650 |
| GPS_P6492 | ERR876651 |
| GPS_P6493 | ERR876652 |
| GPS_P6494 | ERR876653 |
| GPS_P6495 | ERR876654 |
| GPS_P6496 | ERR876655 |
| GPS_P6497 | ERR876656 |
| GPS_P6498 | ERR876657 |
| GPS_P6499 | ERR876658 |
| GPS_P6500 | ERR876659 |
| GPS_P6501 | ERR876660 |
| GPS_P6502 | ERR876661 |
| GPS_P6503 | ERR876662 |
| GPS_P6504 | ERR876663 |
| GPS_P6505 | ERR876664 |
| GPS_P6506 | ERR876665 |
| GPS_P6507 | ERR876666 |
| GPS_P6508 | ERR876667 |
| GPS_P6509 | ERR876668 |
| GPS_P6510 | ERR876669 |
| GPS_P6511 | ERR876670 |
| GPS_P6512 | ERR876671 |
| GPS_P6513 | ERR876672 |
| GPS_P6514 | ERR876673 |
| GPS_P6515 | ERR876674 |
| GPS_P6516 | ERR876675 |
| GPS_P6517 | ERR876676 |
| GPS_P6518 | ERR876677 |
| GPS_P6519 | ERR876678 |
| GPS_P6520 | ERR876679 |
| GPS_P6521 | ERR876680 |
| GPS_P6522 | ERR876681 |
| GPS_P6523 | ERR876682 |
| GPS_P6524 | ERR876683 |
| GPS_P6525 | ERR876684 |
| GPS_P6526 | ERR876685 |
| GPS_P6527 | ERR876686 |
| GPS_P6528 | ERR876687 |
| GPS_P6529 | ERR876688 |
| GPS_P6530 | ERR876689 |
| GPS_P6531 | ERR876690 |
| GPS_P6532 | ERR876691 |
| GPS_P6533 | ERR876692 |
| GPS_P6534 | ERR876693 |
| GPS_P6535 | ERR876694 |
| GPS_P6536 | ERR876695 |
| GPS_P6537 | ERR876696 |
| GPS_P6538 | ERR876697 |
